# Poor lung ultrasound score in shock patients admitted to the ICU is associated with worse outcome

**DOI:** 10.1186/s12890-018-0755-9

**Published:** 2019-01-03

**Authors:** Wanhong Yin, Tongjuan Zou, Yao Qin, Jing Yang, Yi Li, Xueying Zeng, Yan Kang, Chinese Critical Ultrasound Study Group (CCUSG)

**Affiliations:** Department of Critical Care Medicine, West China school of medicine/West China hospital, Sichuan University, 37 Guoxue Avenue, Chengdu, 610041 People’s Republic of China

**Keywords:** Critical care ultrasound, Lung ultrasound score, Shock, ICU, Prognosis

## Abstract

**Background:**

The lung ultrasound score has been regarded as a decent semiquantitative score to measure the lung aeration loss. The score has been proven to be valuable in diagnosing and monitoring lung pathology, but no studies have demonstrated its relationship to the outcome. We aimed to investigate the relationship between the lung ultrasound score and outcome in shock patients in the Intensive Care Unit.

**Methods:**

The data were extracted from the SHOCK-ICU study, a 14-month prospective study of shock patients in the Medical Intensive Care Unit in West China Hospital. A bivariate logistic regression model was established to identify the correlation between the lung ultrasound score on admission and the 28-day mortality. For subsequent analyses, we divided patients into lung ultrasound score quartiles, and survival analysis was performed using Cox stratified survival analysis and regression analysis with the Breslow method of ties.

**Results:**

A total of 175 cases with a completed lung ultrasound exam were included. The mean APACHE II score was 23.7 ± 8.8, and the 28-day mortality was 46.3% (81/175). The multivariate analysis demonstrated that the lung ultrasound score was an independent risk factor for 28-day mortality, as well as the APACHE II score and lactate level. When divided into lung ultrasound score quartiles, after correcting for the APACHE II score, vasoactive use, PaO_2_/FiO_2_, and lactate level, the COX analysis reveals that a higher lung ultrasound score was related to a lower survival rate. Quartile 1 and quartile 2 had a significantly lower hazard ratio versus quartile 4 (OR 0.442[0.215–0.911]; 0.484[0.251–0.934], respectively).

**Conclusions:**

The lung ultrasound score is independently related to the 28-day mortality, as well as the APACHE II score and lactate level, in Intensive Care Unit shock patients. A higher elevated lung ultrasound score on admission is associated with a worse outcome.

**Trial registration:**

The study is registered on Clinical Trials. Trial registration: NCT03082326; retrospectively registered on 3 March 2017.

## Background

Lung ultrasound has been widely used in diagnosing pulmonary diseases including pneumonia, connective tissue diseases and interstitial lung diseases. For patients in the intensive care unit (ICU), more attention is paid to monitoring the development of lung pathologic changes, which guides the therapy [1–9]. Lung insults caused by inflammation, trauma or water increase always lead to infiltration, which results in the loss of lung air. Depending on the severity of the aeration loss and water increase, each part of the lung generates different ultrasound signs upon exam. The lung ultrasound score (LUSS) is the sum of the scores of each exam zone and has been justified as a respectable semiquantitative score to measure the lung aeration loss caused by different lung pathologic changes, such as pneumonia, atelectasis, pleural effusion, and lung oedema [10–12].

Representing the severity of the lung insult, the LUSS can be applied to guide clinical judgement [13–16]. Several studies have demonstrated that the LUSS has been proven to predict post-extubation stress and successfully assess the respiratory effects of antimicrobial therapy in patients with ventilator-associated pneumonia [10, 17, 18]. However, the main weakness in their study was that they offered no attempt to describe the relationship to the outcome. In this paper, we aimed to investigate the value of the LUSS in ICU shock patients and its association with the outcome.

## Methods

### Data collection and design

This was an observation retrospective secondary analysis of a previous study, registered with the number NCT03082326 and approved by the ethics committee of West China Hospital of Sichuan University (2017 (200)). The previous study enrolled 181 consecutive admitted patients who met the criteria for shock and aimed to describe and analyse the physiopathologic characteristics of shock patients assessed by critical care ultrasound on ICU admission. The following are some details of the previous study: right after patients enrolled, the completed lung ultrasound examination was required as part of a complete ultrasound assessment examination. The examinations were performed by a board certificated physician who had completed a full critical care ultrasound (CCUS) training course and had more than a half-year of critical care ultrasonic performance experience. The investigators recorded the ultrasonic data, which were blinded to the treatment team, and assessed the outcome. The data consisted of clinical and ultrasonic variables that were entered into the database after the patient’s discharge or death.

In the current study, we extracted and listed the patients’ demographics, clinical characteristics, prognosis, and the LUSS as part of the indicators in this study. We then established a bivariate logistic regression model to identify the correlation between the LUSS on admission and the 28-day mortality and divided the patients into LUSS quartiles. The COX model was employed to investigate the multiplicative relationship between the predictors and the hazards.

### Lung ultrasound score (LUSS)

**Fig. 1:**
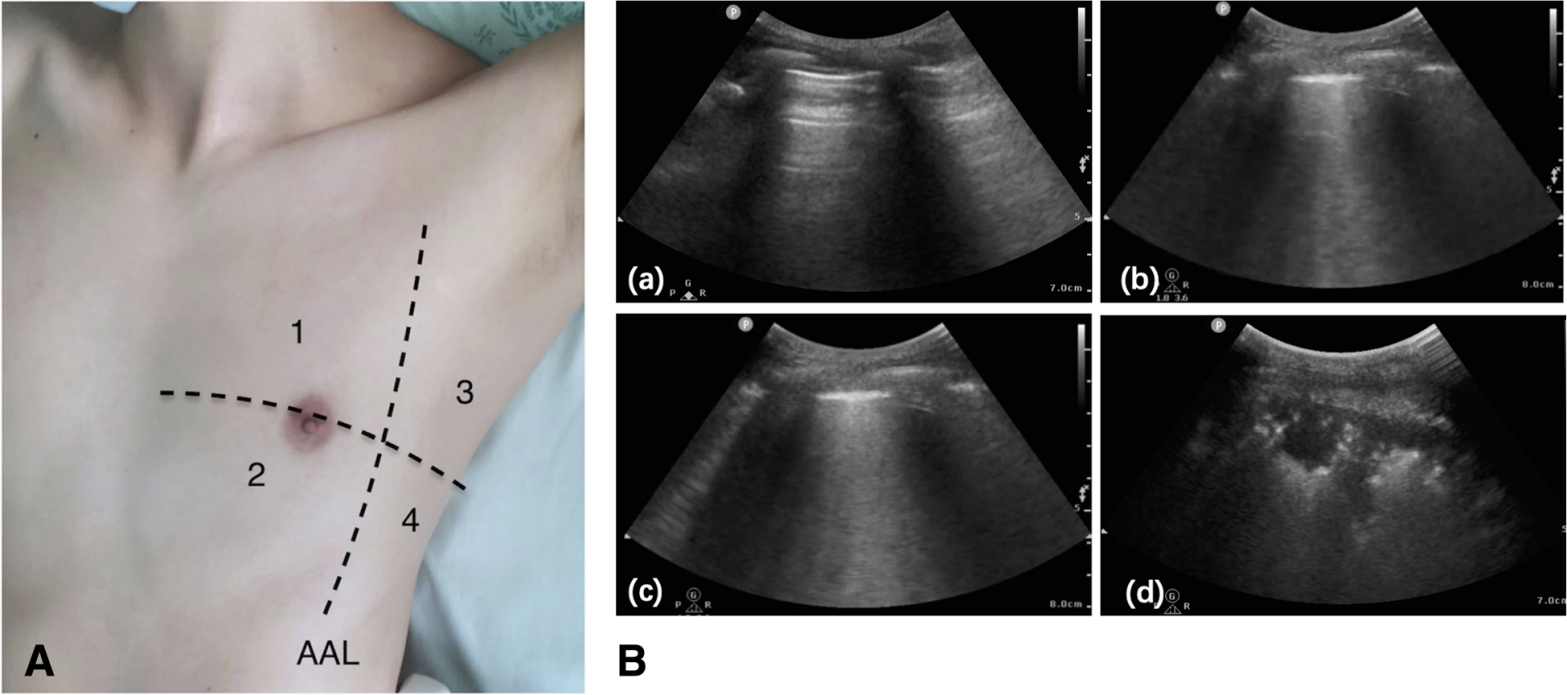
Eight-zone lung ultrasound examination protocol and lung ultrasound pattern. **A**: Each hemithorax is separated into four quadrants: anterior and lateral zones (separated by the anterior axillary lines) with each one divided into upper and lower portions. AAL indicates the anterior axillary line. **B**: Lung ultrasound pattern. (a): A pattern; (b): B1 pattern; (c): B2 pattern; (d): C pattern (lung consolidation).

We used reliable techniques based on the international evidence-based recommendations for point-of-care lung ultrasound [19] that recommended using a complete eight-zone lung ultrasound examination to evaluate the LUSS [12]. The anterior and lateral chest wall were divided into eight areas. Areas 1 and 2 denote the upper anterior and lower anterior chest areas, respectively, and areas 3 and 4 denote the upper lateral and basal lateral chest areas, respectively. For clinical practicability, we adopted the eight-zone examination in the study. Each zone was scored according to the lung ultrasound pattern as follows [10–12] (Fig. 1): the presence of lung sliding with A-lines or fewer than two isolated B-lines, scored 0; when multiple well-defined B-lines (B1-lines) presented, scored 1; the presence of multiple coalescent B-lines (B2-lines), scored 2; and when presented with a tissue pattern characterized by dynamic air bronchograms (lung consolidation), scored 3. The worst ultrasound pattern observed in each zone was recorded and used to calculate the sum of the scores (total score = 24).

### Statistical analysis

The data were analysed using the SPSS 22.0 statistical software. The values were expressed as the means ± standard deviation or median quartiles (first - third quartile) according to their distribution for continuous variables or as counts and percentages for categorical variables. Continuous variables were also expressed as ranges. A bivariate logistic regression model was established, and univariate analysis was undertaken to identify the correlation between the variables and 28-day mortality. The multivariate analysis was conducted to determine whether the LUSS was independently related to 28-day mortality. For subsequent analyses, we divided patients into the LUSS quartile. The survival analysis was performed using Cox stratified survival analysis and regression analysis with the Breslow method of ties to investigate the relationship of the LUSS and 28-day mortality and the trend. Stratification was performed for the LUSS quartiles. PaO_2_/FiO_2_, lactate, and severity of illness are the most prominent confounding variables concerning risk of mortality in shock patients. To account for these variables, we used Cox regression analysis stratified according to the LUSS quartiles and included PaO_2_/FiO_2_, the APACHE II score, lactate level and dose of norepinephrine as covariates. The hazard ratios were calculated relative to quartile 4 of the LUSS using Cox proportional hazards, again controlling for PaO_2_/FiO_2_, the APACHE II score, lactate level and dose of norepinephrine. Hazard ratios are presented with their 95% confidence intervals. Differences in the LUSS between survivors and nonsurvivors were analysed using the Mann-Whitney rank sum test. *P* < 0.05 was considered statistically significant.

## Results

### Demographic and clinical characteristics

A retrospective secondary analysis of the data was performed in the previous study from April 2016 to June 2017, and 6 patients were excluded because of incomplete lung ultrasound examination data, leaving 175 cases (male/female: 108/67) for inclusion in the current study. The mean age was 58.0 ± 18.0 years, while the male to female ratio was 1.6:1. The mean APACHE II score was 23.7 ± 8.8 and ranged from 2 to 50. Upon ICU admission, the average mean arterial pressure (MAP) was 79.5 ± 15.5 mmHg, with a median lactate level of 3.4 (2.0 to 6.8) mmol/L and a range from 1 to 28.2. The LUSS varied from 0 to 22, and the 28-day mortality was 46.3% (81/175). The data are shown in Table 1. As presented in Table 2, the diagnosis on the admission of the study group is listed.

**Table 1:** Demographic and clinical characteristics on admission and outcome of the studied subjects

Variable	Measure	Range
Sex (male/female)	108/67	Not available
Age/years	58.0 ± 18.0	20.0–89.0
APACHE II	23.7 ± 8.8	2.0–50.0
Heart rate	117.6 ± 24.2	70.0–180.0
Mean blood pressure/mmHg	79.5 ± 15.5	43.7–136.3
Urine output per hour	50.0(20.0 to 90.0)	0.0–500.0
Lactate	3.4(2.0 to 6.8)	1.0–28.2
PaO_2_/ FiO_2_	185.0(125.0 to 265.0)	44.0–620.0
Length of mechanical ventilation/hours	167.2(89.0 to 384.0)	0–1405.0
Type of shock/case(%)		
distributive shock	108(61.7)	Not available
hypovolemic shock	52(29.7)	Not available
cardiogenic shock	12(6.9)	Not available
obstructive shock	3(1.7)	Not available
ICU length of stay/d	15.0(7.0 to 28.0)	2.0–138.0
Hospital length of stay/d	23.0(13.0 to 38.0)	2.0–149.0
28-day mortality/%	46.3(81/175)	Not available

*APACHE II* Acute Physiology and Chronic Health Evaluation II, *ICU* intensive care unit

**Table 2:** Admission diagnoses and the proportion

Diagnosis	*n* = 175	%
Respiratory disease	35	20.0%
Severe pneumonia	21	12.0%
AECOPD	5	2.86%
ARDS	7	4.0%
Tracheoesophageal fistula	2	1.14%
Abdominal diseases	51	29.14%
Severe acute pancreatitis	23	13.14%
Gastrointestinal perforation	13	7.43%
Bowel obstruction	6	3.43%
Tumour	3	1.71%
Acute obstructive suppurative cholangitis	6	3.43%
Bloodstream infection	5	2.86%
Subcutaneous infection	5	2.86%
Urinary tract infection	4	2.29%
CNS infection	3	1.71%
Bowel infection	3	1.71%
Infective endocarditis	1	0.57%
Gastrointestinal bleeding	21	12.0%
Arterial aneurysm	4	2.29%
Multiple trauma	2	1.14%
Cardiac arrest	14	8.0%
Heart failure (acute /chronic)	2	1.14%
Myocardial infarction	3	1.71%
Malignant arrhythmia	1	0.57%
High-level spinal cord injury	2	1.14%
Pulmonary embolism	3	1.71%
Pericardial tamponade	1	0.57%
Stroke	9	5.14%
Organ transplantation	6	3.43%

*AECOPD* acute exacerbation of chronic obstructive pulmonary disease, *ARDS* Acute respiratory distress syndrome, *CNS* central nervous system

### Outcomes and multivariate analysis

The most striking result from Table 3 is that the 28-day mortality was significantly correlated with age, the APACHE II score, heart rate, lactate level, urine output, use of vasoactive agents, PaO_2_/FiO_2_ and the LUSS (*p* = 0.011, 0.000, 0.048, 0.000, 0.008, 0.027, 0.031, 0.001, respectively). The multivariate analysis, which referred to the variables with a significant difference in the univariate analysis, demonstrated that the LUSS was an independent risk factor for 28-day mortality, as well as the APACHE II score and lactate level (Table 4).

**Table 3:** Univariate correlation analysis between the LUSS and clinical indexes and 28-day mortality

Indexes	28-day mortality	
	r	*p*
Sex	0.096	0.758
Age	0.022	0.011
APACHE II	0.089	0.000
Heart rate	0.013	0.048
MAP	−0.005	0.597
Lactate	0.158	0.000
Urine output per hour	−0.008	0.008
Vasoactive agents	−1.296	0.027
PO_2_/FiO_2_	−0.003	0.031
LUSS	0.091	0.001

*APACHE II* Acute Physiology and Chronic Health Evaluation II, *MAP* mean arterial pressure, *LUSS* lung ultrasound score

The univariate analysis revealed that age, APACHE II score, heart rate, lactate level, urine output per hour, vasoactive agents, PO_2_/FiO_2,_ and the LUSS were significantly associated with 28-day mortality

**Table 4:** Multivariate analysis of independent risk factors for 28-day mortality

Indexes	28-day mortality		
	*p*	OR	95% CI
Age	0.182	1.015	0.993–1.036
APACHE II	0.025	1.054	1.007–1.103
Heart rate	0.157	1.011	0.996–1.026
Lactate	0.003	1.129	1.043–1.222
Urine output per hour	0.067	0.994	0.988–1.000
Use of vasoactive agents	0.117	0.360	0.101–1.291
PO_2_/FiO_2_	0.648	1.001	0.997–1.004
LUSS	0.029	1.074	1.007–1.146

*APACHE II* Acute Physiology and Chronic Health Evaluation II, *MAP* mean arterial pressure, *LUSS* lung ultrasound score

The LUSS was an independent risk factor for 28-day mortality, as well as the APACHE II score and lactate level

**Fig. 2:**
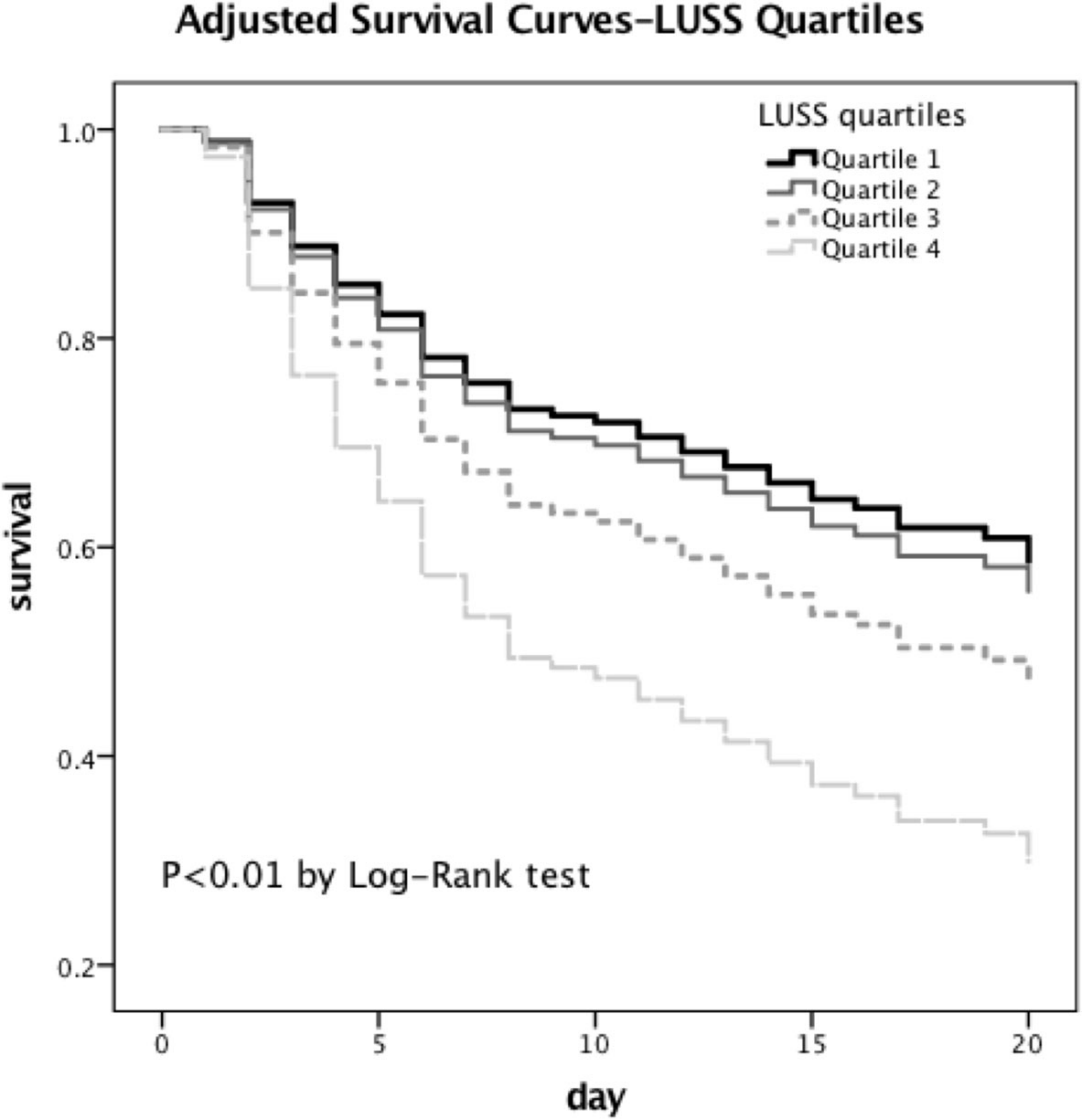
Survival analysis for the four LUSS quartiles. After correcting for PaO_2_/ FiO_2_, the APACHE II score, vasoactive use and lactate, the survival analysis reveals that a higher LUSS was related to a lower survival rate.

Patients were then divided into LUSS quartiles; the characteristics are shown in Table 5. After correcting for PaO_2_/FiO_2_, the APACHE II score, vasoactive use, and lactate level, the survival analysis revealed that a higher LUSS was related to a lower survival rate (Fig. 2). As disclosed in Table 6, quartile 1 and quartile 2 have a significantly lower hazard ratio versus quartile 4 (OR 0.442[0.215–0.911]; 0.484[0.251–0.934], respectively).

**Table 5:** The characteristic of each LUSS quartile

Variable (*n*)	Quartile 1 (43)	Quartile 2 (47)	Quartile 3 (42)	Quartile 4 (43)	*p*
Sex (male/female)	27/16	30/17	25/17	26/17	0.974
Age	55.8 ± 17.9	55.7 ± 19.1	63.6 ± 17.5	57.1 ± 16.8	0.136
APACHE II	20.0 ± 6.9	22.2 ± 7.8	27.5 ± 11.1	25.5 ± 7.4	0.000^#^
Heart rate	112.5 ± 27.0	116.9 ± 21.6	121.5 ± 24.7	119.7 ± 23.1	0.340
Mean arterial pressure	82.5 ± 16.6	78.2 ± 14.4	78.9 ± 16.5	78.4 ± 14.5	0.523
Urine output per hour	50.0(20.0–80.0)	50.0(20.0–120.0)	40.0(4.3–72.5)	60.0(30.0–90.0)	0.291
Lactate	2.2(1.7–4.2)	3.2(2.1–7.5)	3.5(2.2–5.8)	4.0(2.4–9.6)	0.014^#^
PaO_2_/ FiO_2_	262.3 ± 141.1	253.0 ± 109.4	177.8 ± 110.1	153.2 ± 69.2	0.000^#^
Length of mechanical ventilation/hours	172.0(74.0–426.0)	155.0(84.0–401.1)	173.0(112.3–343.1)	168.0(89.0–316.0)	0.962
LUSS	1.9 ± 1.6	7.2 ± 1.3	11.6 ± 1.3	17.5 ± 2.0	0.000^#^
ICU length of stay/d	17.0(9.0–38.0)	15.0(9.0–31.0)	13.5(6.0–30.0)	11.0(5.0–21.0)	0.138
Hospital length of stay/d	26.0(17.0–51.0)	28.0(13.0–43.0)	23.0(13.0–31.3)	17.0(5.0–35.0)	0.038^#^
28-day mortality/%(n/N)	34.9(15/43)	34.0(16/47)	50.0(21/42)	67.4(29/43)	0.005^#^

*APACHE II* Acute Physiology and Chronic Health Evaluation II, *MAP* mean arterial pressure, *LUSS* lung ultrasound score, *ICU*: intensive care unit

#:APACHE II score, lactate, PaO_2_/ FiO_2_, LUSS, hospital length of stay and 28-day mortality showed significant differences in four LUSS quartiles

**Table 6:** Hazard ratio for 28-day mortality according to LUSS quartiles

LUSS Group	Hazard Ratio versus Quartile 4 OR (95% CI)	*p*
Quartile 1	0.442(0.215–0.911)	0.027
Quartile 2	0.484(0.251–0.934)	0.031
Quartile 3	0.632(0.348–1.149)	0.132

*LUSS* lung ultrasound score

## Discussion

The main finding of our study is that the LUSS was considered to be an independent risk factor closely related to 28-day mortality, as well as the APACHE II score and lactate level (*p* = 0.029, 0.025, 0.003; respectively). The result demonstrated that a higher LUSS was significantly associated with an increased 28-day mortality (Fig. 2). To our knowledge, no similar study has been published previously.

Shock is a common but severe condition in patients in the intensive care unit and is regarded as one of the leading causes of death [20, 21]. The mean arterial pressure and lactate level are two valuable variables used to describe the severity of shock. Moreover, these two factors serve as the clinical resuscitation goals as well, since they are considered to be highly associated with the outcome [22–25]. However, direct or indirect pulmonary insults are common complications in shock patients and are closely associated with mortality [26–28]. Previously, PaO_2_/FiO_2_ was widely used to represent the result of lung damage; however, it cannot reveal the pathophysiological changes of the lung and guide the therapeutic plan accurately. In this aspect, the LUSS has the ability to show a clear advantage over PaO_2_/FiO_2_ because it not only describes the severity of lung damage but also presents the detailed and visualized pathophysiological changes during the examination.

The results of the study support that the value of the LUSS was equal in importance to the lactate level in shock patients, which means that the caregivers should focus on lung protection and settle on specific lung pathology treatment plans compared to previous protocols that only focused on resuscitating the circulation. The LUSS deserves consideration as a recommendation to guide the therapy and titration in future guidelines.

Although being of value to semiquantitatively measure the lung damage, the LUSS still has expansions compared to other variables such as the oxygenation index and extravascular lung water index (ELWI). The latter two indexes represent the severity of the lung damage and the risk of fluid overload and prevent excessive volume expansion [26, 27, 29]. Unfortunately, these indexes neglect to reveal the pathophysiological changes of the lung and the reason for the damage. When determining the LUSS, each lung region of interest is examined, and the pathophysiological changes are described with different ultrasonic signs [30–32]. For instance, The A-lines reflect regular lung aeration of the examined zone, while acutely, the presence of B lines may reveal interstitial lung oedema. These natural attributes of the LUSS enhance its application value. Supportively, in the multivariate analysis of our study, the LUSS was presented as an independent indicator of outcome compared with PaO_2_/FiO_2_. Accompanied by the other ultrasonic signs, we could acquire more information on each part of the lung, which is conducive for guiding the treatment such as diuresis, recruitment manoeuvres, pleural effusion drainage, and sputum drainage [4, 5, 19, 33, 34]. Furthermore, with the successive LUSS record during the therapeutic process, we can evaluate the effect of the treatment [10, 18, 35, 36]. In summary, the LUSS has the capacity to serve as a better index to monitor the lung and guide the treatment in the clinical practice setting.

Initially, we thought that the COX analysis might provide more interesting results. However, a more careful inspection revealed that quartile 3 and quartile 4 did not show a significant difference in 28-day mortality. This result may be because of the following reasons: first, quartile 3 had already represented a high severity (11.6 ± 1.3); second, this sample size was slightly lower than the value we expected, and there is certainly room for improvement.

We are aware that our research may have some limitations. The first is that the data were extracted from a single teaching hospital; therefore, the results may not be generalizable to other types of institutions. The second limitation is that the original prospective study did not aim to investigate the association of the LUSS with the outcome, which may reduce the power of the design of the present study. The third limitation is that the mortality was high (46.3%) in our study compared with that in other studies [19, 23]. Of note, in our study we included all types of shock, and as a large medical centre in west China, the patients who were transferred to our centre usually had a more severe overall condition (APACHE II 23.7 ± 8.8). These factors might affect the representativeness of the study to a generally healthier patient population. The limitations highlight the difficulty of data analysis and application. However, there were well-designed protocols of the CCUS exam to be followed including lung ultrasound prior to the original study, and the data were recorded and entered independently by the investigator, which makes the data reliable and valuable. Nevertheless, the nature of the retrospective analysis limits our ability to determine a causal relationship between the LUSS and the outcomes. Although the conclusions were proved by the results of different statistical means, that is, univariate analysis, multivariate analysis and COX analysis, a well-designed prospective study focusing on the LUSS is required in future projects.

## Conclusions

In conclusion, based on our study, the LUSS is independently related to the 28-day mortality as well as the APACHE II score and lactate levels in ICU shock patients. An elevated LUSS on admission is associated with a worse outcome. Hence, this finding requires the same concern as other indicators on hospital admission as well as the treatment.

## Abbreviations

AECOPD: Acute exacerbation of chronic obstructive pulmonary disease
APACHE II: Acute Physiology and Chronic Health Evaluation II
ARDS: Acute Respiratory Distress Syndrome
CNS: Central nervous system.
ELWI: Extravascular lung water index
ICU: Intensive care unit
LUSS: Lung ultrasound score
MAP: Mean arterial pressure

## References

[1] Via G, Storti E, Gulati G, Neri L, Mojoli F, Braschi A (2012). Lung ultrasound in the ICU: from diagnostic instrument to respiratory monitoring tool. Minerva Anestesiol.

[2] Le NA, Mongodi S, Philippart F, Bouhemad B (2016). Thoracic ultrasound: potential new tool for physiotherapists in respiratory management. A narrative review. J Crit Care.

[3] Liccardo B, Martone F, Trambaiolo P, Severino S, Cibinel GA, D’Andrea A (2016). Incremental value of thoracic ultrasound in intensive care units: indications, uses, and applications. World J Radiol.

[4] Bataille B, Riu B, Ferre F, Moussot PE, Mari A, Brunel E, Ruiz J, Mora M, Fourcade O, Genestal M (2014). Integrated use of bedside lung ultrasound and echocardiography in acute respiratory failure: a prospective observational study in ICU. Chest.

[5] Wang XT, Liu DW, Zhang HM, Chai WZ (2014). Integrated cardiopulmonary sonography: a useful tool for assessment of acute pulmonary edema in the intensive care unit. J Ultrasound Med.

[6] Frankel HL, Kirkpatrick AW, Elbarbary M, Blaivas M, Desai H, Evans D, Summerfield DT, Slonim A, Breitkreutz R, Price S (2015). Guidelines for the appropriate use of bedside general and cardiac ultrasonography in the evaluation of critically ill patients-part I: general ultrasonography. Crit Care Med.

[7] Lichtenstein DA, Lascols N, Mezière G, Gepner A (2004). Ultrasound diagnosis of alveolar consolidation in the critically ill. Intensive Care Med.

[8] Parlamento S, Copetti R, Bartolomeo SD (2009). Evaluation of lung ultrasound for the diagnosis of pneumonia in the ED. Am J Emerg Med.

[9] Tardella M, Di Carlo M, Carotti M, Filippucci E, Grassi W, Salaffi F (2018). Ultrasound B-lines in the evaluation of interstitial lung disease in patients with systemic sclerosis: cut-off point definition for the presence of significant pulmonary fibrosis. Medicine.

[10] Soummer A, Perbet S, Brisson H, Arbelot C, Constantin JM, Lu Q, Rouby JJ, Group LUS (2012). Ultrasound assessment of lung aeration loss during a successful weaning trial predicts postextubation distress*. Crit Care Med.

[11] Caltabeloti FP, Monsel A, Arbelot C, Brisson H, Lu Q, Gu WJ, Zhou GJ, Auler JOC, Rouby JJ (2014). Early fluid loading in acute respiratory distress syndrome with septic shock deteriorates lung aeration without impairing arterial oxygenation: a lung ultrasound observational study. Crit Care.

[12] Volpicelli G, Mussa A, Garofalo G, Cardinale L, Casoli G, Perotto F, Fava C, Frascisco M (2006). Bedside lung ultrasound in the assessment of alveolar-interstitial syndrome. Am J Emerg Med.

[13] Sperandeo M, Carnevale V, Muscarella S, Sperandeo G, Varriale A, Filabozzi P, Piattelli ML, D'Alessandro V, Copetti M, Pellegrini F (2011). Clinical application of transthoracic ultrasonography in inpatients with pneumonia. Eur J Clin Investig.

[14] Chinardet B, Brisson H, Arbelot C, Langeron O, Rouby JJ, Lu Q (2016). Ultrasound assessment of lung consolidation and reaeration after pleural effusion drainage in patients with acute respiratory distress syndrome: a pilot study. Acta Anaesthesiol Belg.

[15] Narasimhan M, Koenig SJ, Mayo PH (2016). A whole-body approach to point of care ultrasound. Chest.

[16] Bouhemad B, Brisson H, Leguen M, Arbelot C, Lu Q, Rouby JJ (2011). Bedside ultrasound assessment of positive end-expiratory pressure–induced lung recruitment. Am J Respir Crit Care Med.

[17] Zagli G, Cozzolino M, Terreni A, Biagioli T, Caldini AL, Peris A (2014). Diagnosis of ventilator-associated pneumonia: a pilot, exploratory analysis of a new score based on procalcitonin and chest echography. Chest.

[18] Bouhemad B, Liu ZH, Arbelot C, Mao Z, Ferarri F, Guen ML, Girard M, Qin L, Rouby JJ (2010). Ultrasound assessment of antibiotic-induced pulmonary reaeration in ventilator-associated pneumonia. Crit Care Med.

[19] Volpicelli G, Elbarbary M, Blaivas M, Lichtenstein DA, Mathis G, Kirkpatrick AW, Melniker L, Gargani L, Noble VE, Via G (2012). International evidence-based recommendations for point-of-care lung ultrasound. Intensive Care Med.

[20] Vincent JL, De BD (2013). Circulatory shock. N Engl J Med.

[21] Rhodes A, Evans LE, Alhazzani W, Levy MM, Antonelli M, Ferrer R, Kumar A, Sevransky JE, Sprung CL, Nunnally ME (2017). Surviving Sepsis campaign: international guidelines for Management of Sepsis and Septic Shock: 2016. Intensive Care Med.

[22] Rivers E, Nguyen B, Havstad S, Ressler J, Muzzin A, Knoblich B, Peterson E, Tomlanovich M (2001). Early goal-directed therapy in the treatment of severe sepsis and septic shock. N Engl J Med.

[23] Vincent JL, Rhodes A, Perel A, Martin GS, Rocca GD, Vallet B, Pinsky MR, Hofer CK, Teboul JL, Boode WPD (2011). Clinical review: update on hemodynamic monitoring - a consensus of 16. Crit Care.

[24] Cecconi M, De Backer D, Antonelli M, Beale R, Bakker J, Hofer C, Jaeschke R, Mebazaa A, Pinsky MR, Teboul JL (2014). Consensus on circulatory shock and hemodynamic monitoring. Task force of the European Society of Intensive Care Medicine. Intensive Care Med.

[25] Nguyen HB, Rivers EP, Knoblich BP, Jacobsen G, Muzzin A, Ressler JA, Tomlanovich MC (2004). Early lactate clearance is associated with improved outcome in severe sepsis and septic shock. Crit Care Med.

[26] Chung FT, Lin HC, Kuo CH, Yu CT, Chou CL, Lee KY, Kuo HP, Lin SM (2010). Extravascular lung water correlates multiorgan dysfunction syndrome and mortality in sepsis. PLoS One.

[27] Kuzkov VV, Kirov MY, Sovershaev MA, Kuklin VN, Suborov EV, Waerhaug K, Bjertnaes LJ (2006). Extravascular lung water determined with single transpulmonary thermodilution correlates with the severity of sepsis-induced acute lung injury. Crit Care Med.

[28] Martin GS, Bernard GR (2001). Airway and lung in sepsis. Intensive Care Med.

[29] Fernández-Mondéjar E, Guerrero-López F, Colmenero M (2007). How important is the measurement of extravascular lung water?. Curr Opin Crit Care.

[30] Via G, Lichtenstein D, Mojoli F, Rodi G, Neri L, Storti E, Klersy C, Iotti G, Braschi A (2010). Whole lung lavage: a unique model for ultrasound assessment of lung aeration changes. Intensive Care Med.

[31] Lichtenstein DA (2015). BLUE-protocol and FALLS-protocol: two applications of lung ultrasound in the critically ill. Chest.

[32] Lichtenstein DA, Mezière GA (2008). Relevance of lung ultrasound in the diagnosis of acute respiratory failure: the BLUE protocol. Chest.

[33] Salem R, Vallee F, M, Mebazaa A: Hemodynamic monitoring by echocardiography in the ICU: the role of the new echo techniques. Curr Opin Crit Care 2008, 14(5):561–568. 10.1097/MCC.0b013e32830e6d81 18787450

[34] Jensen MB, Larsen KM, Schmidt MB (2004). Transthoracic echocardiography for cardiopulmonary monitoring in intensive care. Eur J Anaesthesiol.

[35] Theerawit P, Tomuan N, Sutherasan Y, Kiatboonsri S (2014). Transthoracic ultrasound assessment of B-lines for identifying the increment of extravascular lung water in shock patients requiring fluid resuscitation. Indian J Crit Care Med.

[36] Shyamsundar M, Attwood B, Keating L, Walden AP (2013). Clinical review: the role of ultrasound in estimating extra-vascular lung water. Crit Care.

